# Dual activity of *Serratia marcescens* Pt-3 in phosphate-solubilizing and production of antifungal volatiles

**DOI:** 10.1186/s12866-021-02434-5

**Published:** 2022-01-13

**Authors:** Andong Gong, Gaozhan Wang, Yake Sun, Mengge Song, Cheelo Dimuna, Zhen Gao, Hualing Wang, Peng Yang

**Affiliations:** 1 Henan Key Laboratory of Tea Plant Biology, College of Life Science, Xinyang Normal University, Xinyang, 464000 People’s Republic of China; 2 College of Forestry, Hebei Agricultural University, Baoding, 071000 People’s Republic of China

**Keywords:** *Serratia marcescens*, Phosphate solubilizing, Antifungal activity, Dimethyl disulfide

## Abstract

**Background:**

Soil fertility decline and pathogen infection are severe issues for crop production all over the world. Microbes as inherent factors in soil were effective in alleviating fertility decrease, promoting plant growth and controlling plant pathogens et al. Thus, screening microbes with fertility improving and pathogen controlling properties is of great importance to humans.

**Results:**

Bacteria Pt-3 isolated from tea rhizosphere showed multiple functions in solubilizing insoluble phosphate, promoting plant growth, producing abundant volatile organic compounds (VOCs) and inhibiting the growth of important fungal pathogens in vitro. According to the 16S rRNA phylogenetic and biochemical analysis, Pt-3 was identified to be *Serratia marcescens*. The solubilizing zone of Pt-3 in the medium of lecithin and Ca_3_(PO_4_)_2_ was 2.1 cm and 1.8 cm respectively. In liquid medium and soil, the concentration of soluble phosphorus reached 343.9 mg.L^− 1^, and 3.98 mg.kg^− 1^, and significantly promoted the growth of maize seedling, respectively. Moreover, Pt-3 produced abundant volatiles and greatly inhibited the growth of seven important phytopathogens. The inhibition rate ranged from 75.51 to 100% respectively. Solid phase micro-extraction coupled with gas chromatography tandem mass spectrometry proved that the antifungal volatile was dimethyl disulfide. Dimethyl disulfide can inhibit the germination of *Aspergillus flavus*, and severely destroy the cell structures under scanning electron microscopy.

**Conclusions:**

*S. marcescens* Pt-3 with multiple functions will provide novel agent for the production of bioactive fertilizer with P-solubilizing and fungal pathogens control activity.

## Background

China as the most populous country is a giant producer and consumer of crops in the world. Improving soil fertility and increasing crop yield are necessary to local people. Phosphorus (P) ranked as the second most important macro-nutrient can promote plant growth and facilitate the absorption of N, K, Mg and other nutrients for plants [1]. However, the available P was rare due to its poor solubility in soil [2, 3]. More than 74% of the lands were deficient in P [4]. Hence, the P supplement to plants was mostly relying on the application of chemical P fertilizer. From 1949 to 1992, it was estimated that the accumulative amount of P fertilizer applied to soils reached 3.4 × 10^7^ tons in China, of which about 2.6 × 10^7^ tons was fixed by metal ions [5]. Chemical fertilizers further aggravated the mineralization pollution of soils such as acidification, hardening and fertility reduction et al. [6–8]. Hence, the most efficient and environmental method to solve soil problem was to solubilize phosphorite, and increase the content of available P.

Microorganisms with P solubilizing activity are considered as major agents in alleviating soil mineralization problems [7, 9]. They are capable of turning insoluble P mineral into the soluble P and increased P content in soil [10]. Till now, several kinds of bacteria have been proved efficient in promoting P solubilizing such as *Aspergillus niger* [11], *Pseudomonas* sp. [12]. *Actinomycetes* sp., *Bacillus* sp. [13], *Serratia* sp. [7] and *Burkholderia pyrrocinia* [14]. Among these microbes, *Serratia* species as gram-negative bacteria was widely distributed in soil [15]. They were well known for the degradation of chitin by releasing of chitinase [16], as well as soilborne pathogens control activity [17]. Additionally, *Serratia marcescens* CTM50650, NBRI1213 and GPS-5 showed active P-solubilizing activity in medium and liquid suspension [7, 18, 19]. But, these strains only showed single activity which may limit the broad applications of them. In current study, we isolated bacteria *Serratia marcescens* Pt-3 with dual functions of P solubilizing and antifungal activity. The objectives of our study are to 1) evaluate the P-solubilizing activity of Pt-3 in the media, liquid solution and soils; 2) determine the antagonistic activity against different pathogens, 3) identified the predominant volatile antifungal compounds from Pt-3, and elucidated the inhibitory mechanism.

## Results

### Screening of bacteria with phosphate solubilizing activity

**Fig. 1:**
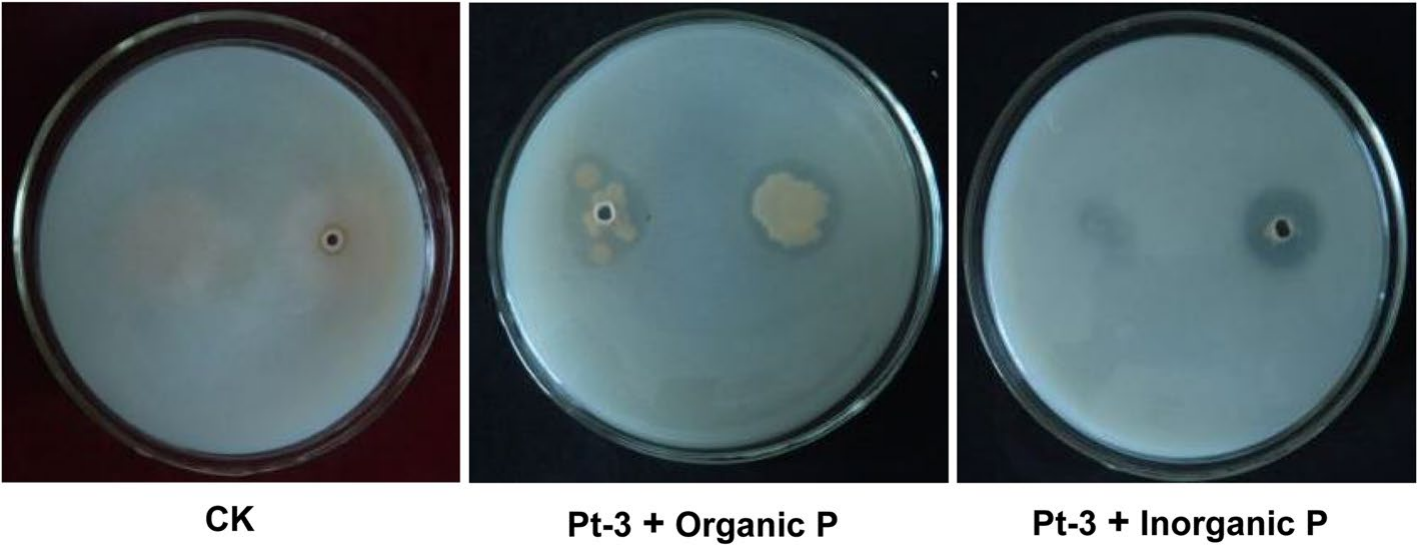
Solubilizing activity of Pt-3 in organic and inorganic Phosphate medium. CK, Phosphate medium without bacteria inoculation; Pt-3 & Organic P, bacteria Pt-3 inoculated in the organic Phosphate (soybean lecithin) medium; Pt-3 & Organic P, Pt-3 inoculated in the inorganic Phosphate (Ca_3_(PO_4_)_2_) medium

To screen microbe with P-solubilizing activity, 893 bacteria were isolated from tea rhizosphere by serial dilution method. Among these bacteria, 9 strains could solubilize inorganic phosphate (Ca_3_(PO_4_)_2_), and 5 strains can solubilize organic phosphate (lecithin) in solid medium. Among these bacteria, strain Pt-3 showed valid activity both in organic and inorganic phosphate medium. A clear and wide halo zone was formed around the clone of Pt-3 in each medium (Fig. 1). And the diameter of halo zone in organic P medium was 2.1 cm. The P solubilization index (PSI) is 1.4. In inorganic media, the halo zone diameter was 1.8 cm, the PSI was 3.6.

### Phosphate solubilizing activity of Pt-3 in liquid and soil conditions

**Fig. 2:**
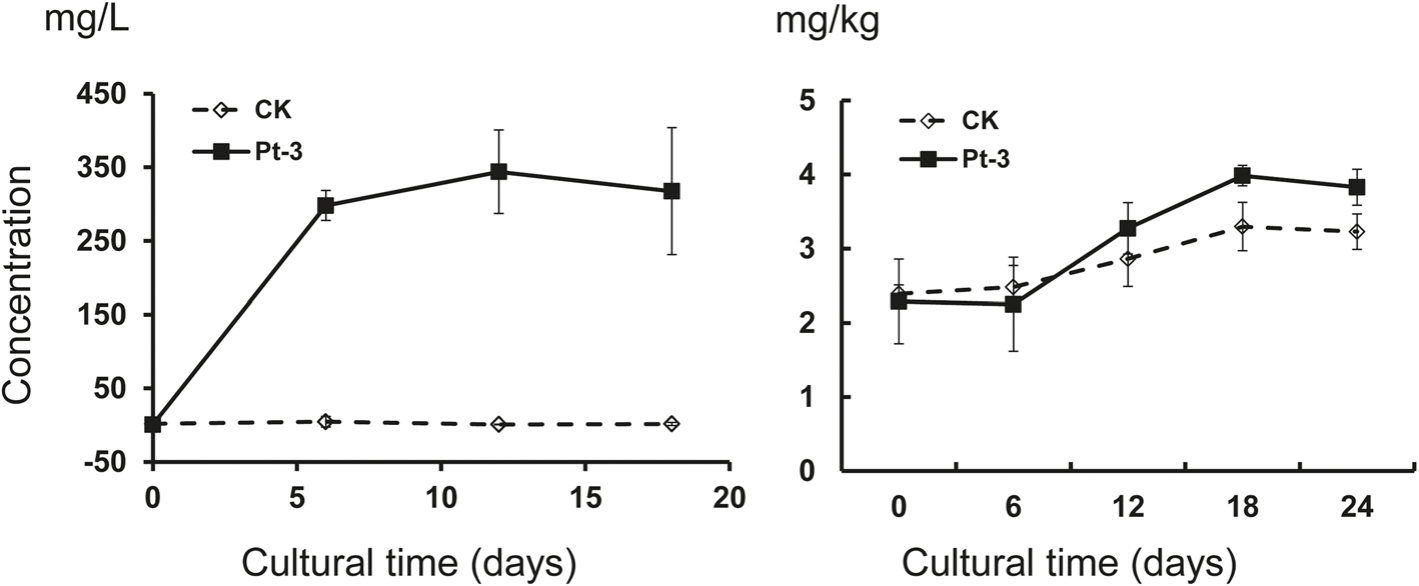
Phosphate solubilizing activity of Pt-3 against Ca_3_(PO_4_)_2_ under liquid and soil conditions. **a** liquid medium of insoluble Ca_3_(PO_4_)_2_ inoculated with Pt-3 and cultured at 30 °C and 150 rpm for 18 days; **b** soil containing Ca_3_(PO_4_)_2_ inoculated with Pt-3 and placed at 30 °C for 24 days

Pt-3 could also promote the solubilizing of P in liquid medium. In the treatment without Pt-3, few PO_4_^3−^ was detected in the suspension, and the concentration showed no changes during 20 days. When bacteria Pt-3 was added into the solution, insoluble Ca_3_(PO_4_)_2_ can be transformed to soluble PO_4_^3−^ and the concentration was increased dramatically during 20 days. In the first 6 days, the concentration of PO_4_^3−^ increased quickly from 0.7 to 343 mg.L^− 1^, then the concentration was stable around 300 mg.L^− 1^ (Fig. 2).

In soil condition, Pt-3 also showed valid P solubilizing activity. The concentration of soluble PO_4_^3−^ was increased from 2.2 to 3.9 mg.kg^− 1^, and stable at 3.8 mg.kg^− 1^ in the 18th days. In control treatment, the soluble PO_4_^3−^ showed less change ranged between 2.3 to 3.2 mg.kg^− 1^ and stable at 3.2 mg.kg^− 1^ in the 18th days. Compared to control, we could clearly observe that the concentration of PO_4_^3−^ in Pt-3 inoculation was equal to control treatment at the beginning. Then the concentration increased higher in Pt-3 treatment during the 6th to 24th days (Fig. 2). These results showed that Pt-3 can solubilize insoluble Ca_3_(PO_4_)_2_ in liquid and soil conditions.

### Pt-3 promoting the growth of maize seedling

Based on the high P solubilizing efficiency of Pt-3, we conducted a 30-day maize growing experiment under atmospheric conditions. The results clearly proved that Pt-3 could promote the growth of maize seedling compared to control treatment. The index of plant shoot leaf length and dry weight in Pt-3 treatment was significantly higher than that in control treatment (Table 1). Additionally, the index of leaf width (2.55 ± 0.08 cm), root length (4.95 ± 0.15 cm) and number of leaves (5 per plant) for Pt-3 were also better than control treatment.

**Table 1:** Effect of *Serratia marcescens* Pt-3 on the growth of maize seedling

Growth parameter	Control	Pt-3
Plant height (cm)	9.40 ± 0.20	14.00 ± 0.30*
Leaf length (cm)	6.20 ± 0.56	9.55 ± 0.60*
Leaf width (cm)	2.27 ± 0.03	2.55 ± 0.08
Root length	4.78 ± 0.04	4.95 ± 0.15
Number of leaves per plant	4.00 ± 0.00	5.00 ± 0.00
Stem diameter (cm)	0.57 ± 0.01	0.65 ± 0.03
Plant dry weight (g/plant)	0.83 ± 0.00	1.65 ± 0.14*

Asterisks (*) mean significant differences compared to control at *p* < 0.05

### Molecular and biochemical characters of strain Pt-3

The single clone of Pt-3 was picked and used for biochemical analysis through MicroStation™ system. The results clearly proved that strain Pt-3 as gram negative bacteria showed positive reaction in the cultural cells of ten carbon sources including glucose, mannitol, maltose and sucrose et al. But it can not grow at the presence of lactose, phenyalanine respectively. It also showed positive reaction at high salt conditions (1 to 8% NaCl), pH 5.0 and 7.0, as well as different antibiotics (streptomycin, lincomycin, vancomycin et al.) (Table 2). These results were consistent with the biochemical characters of *S. marcescens* in MicroStation database.

**Table 2:** Biochemical and physiological analysis of strain Pt-3

	***S. marcescens***	Pt-3
**Carbon Utilization**		
Lactose	–	–
Glucose	+	+
Maltose	+	+
Mannitol	+	+
Sucrose	+	+
Citric acid production	+	+
Gelatin hydrolysis	+	+
Nitric acid	+	+
Lysine decarboxylase	+	+
Lipase	+	+
Gram stain	–	–
Phenyalanine	–	–
Catalase production	+	+
**NaCl tolerance**		
1% NaCl	++	++
4% NaCl	++	++
8% NaCl	+	+
**pH tolerance**		
pH 5	+	++
pH 7	++	++
**Antibiotic resistance**		
Streptomycin	+	+
Lincomycin	++	++
Vancomycin	+	+
Rifamycin SV	++	++
Chloraphenicol	+	+
Gentamycin	+	+
Ampicillin	++	+

*S. marcescens* was the standard strain in Biolog GenIII Microstation system. ++: strong positive reaction; +: positive reaction; −: negative reaction

**Fig. 3:**
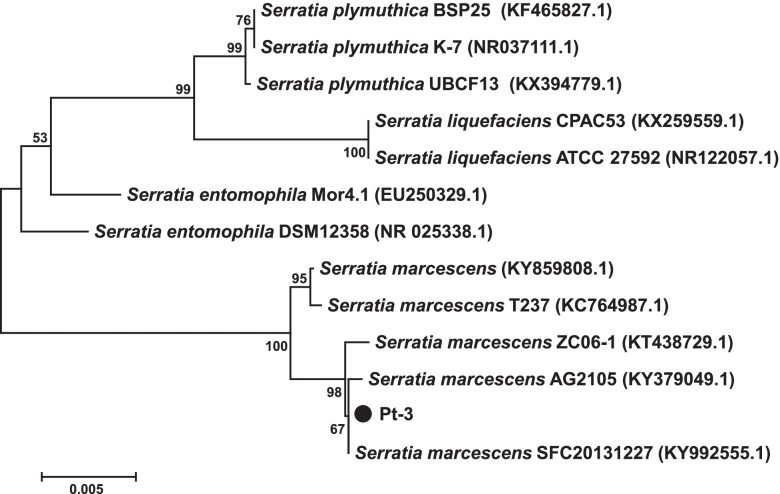
Phylogenetic tree of Pt-3 and homologous strains based on 16S rRNA sequences

16S rRNA sequence was amplified from the genome of Pt-3. The sequence was aligned in GenBank database, which showed high similarity to *Serratia* sp. such as *S. plymuthica*, *S. liquefaciens*, *S. entomophila*, *S. marcescens*. The 16S rRNA sequences of homologous strains and Pt-3 were used to construct phylogenetic tree (Fig. 3). The phylogenetic analysis proved that Pt-3 was homologous to *S. marcescens*. They were classified into same clade with *S. marcescens* (KT438729.1, KY379049.1, KY992555.1). Hence, we could deduce that strain Pt-3 was *S. marcescens* based on 16S rRNA and biochemical analysis.

### Antifungal activity of VOCs from Pt-3

Pt-3 showed broad antifungal activity against different fungi without direct contact. In face-to-face dual cultural tests, the growth of eight important fungal pathogens was all greatly inhibited by Pt-3. The inhibitory rate against *F. graminearum* was 100%, and against other six pathogens (*Magnaporthe oryzae, B. cinerea, A. flavus, A. fumigatus, Colletotrichum graminicola, A. alternata*) the inhibitory rate ranged from 75.51 to 97.83% respectively (Table 3). We can deduce that Pt-3 can produce some antifungal VOCs, spread quickly and inhibit the growth of co-cultured fungal strains.

**Table 3:** Broad spectrum antifungal activity of volatiles from strain Pt-3

Pathogens	Inhibitory rate (%)
*F. graminearum*	100.00 ± 0.00
*Magnaporthe oryzae*	97.83 ± 0.02
*B. cinerea*	92.86 ± 0.04
*A. flavus*	90.83 ± 0.04
*A. fumigatus*	89.30 ± 0.12
*Colletotrichum graminicola*	80.00 ± 0.00
*A. alternata*	75.51 ± 0.32

**Fig. 4:**
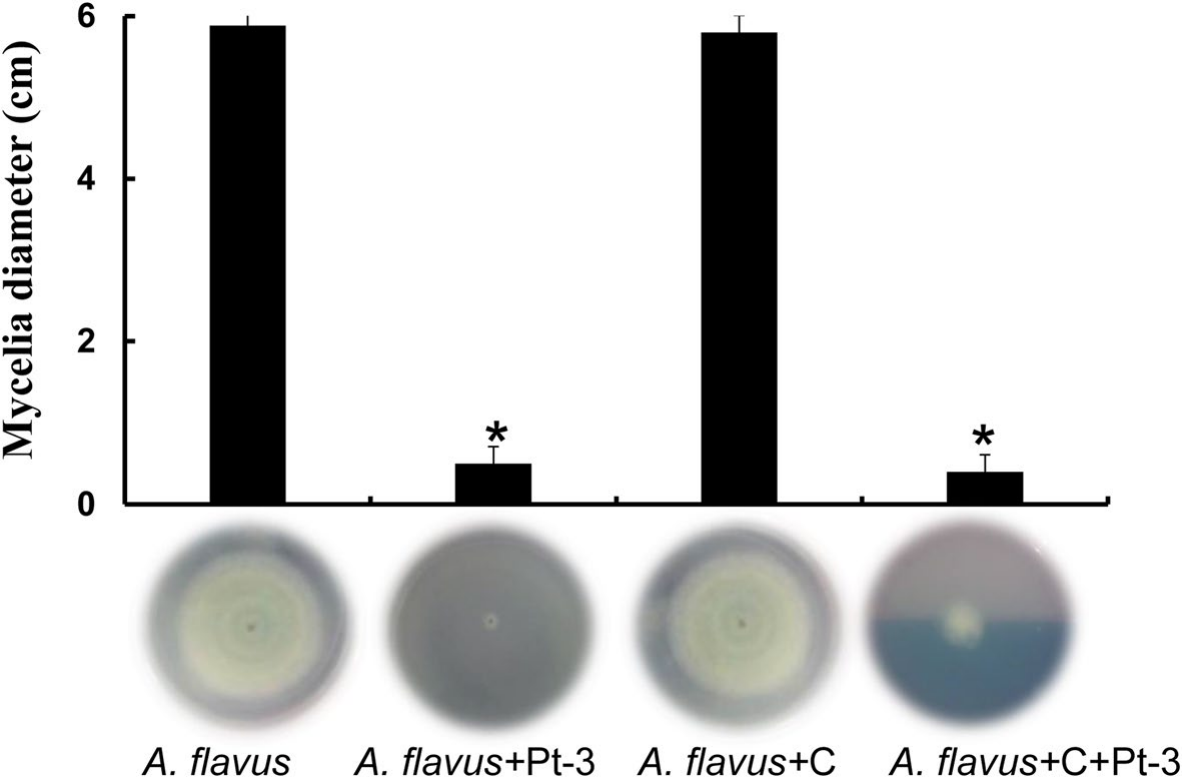
Inhibitory activity of volatiles from Pt-3 against *A. flavus* affected by active charcoal in sealed airspace. Mycelia of *A. flavus* cultured in PDA medium (*A. flavus*) challenged with bacteria Pt-3 spread on NA medium (*A. flavus* + Pt-3) with the presence of active charcoal (*A. flavus* + C + Pt-3). *A. flavus* on PDA challenged with active charcoal was used as control (*A. flavus* + C)

To further prove the production of antifungal VOCs from Pt-3, active charcoal was added into the experiments. The diameter of *A. flavus* mycelia on PDA plate was 6 cm 5 dpi. Active charcoal added into the tests, the growth of *A. flavus* showed no difference. When Pt-3 was added into the tests, the growth of *A. flavus* was greatly inhibited, the diameter was 0.7 cm. But the inhibition activity of Pt-3 was weakened when active charcoals added into Pt-3 and *A. flavus* treatment. These results proved that Pt-3 can produce volatile and inhibit the growth of fungal strains, and active charcoal as adsorbent can absorb some VOCs from Pt-3, and weakened the inhibitory activity of Pt-3 (Fig. 4).

### Identification of antifungal VOCs from Pt-3

**Fig. 5:**
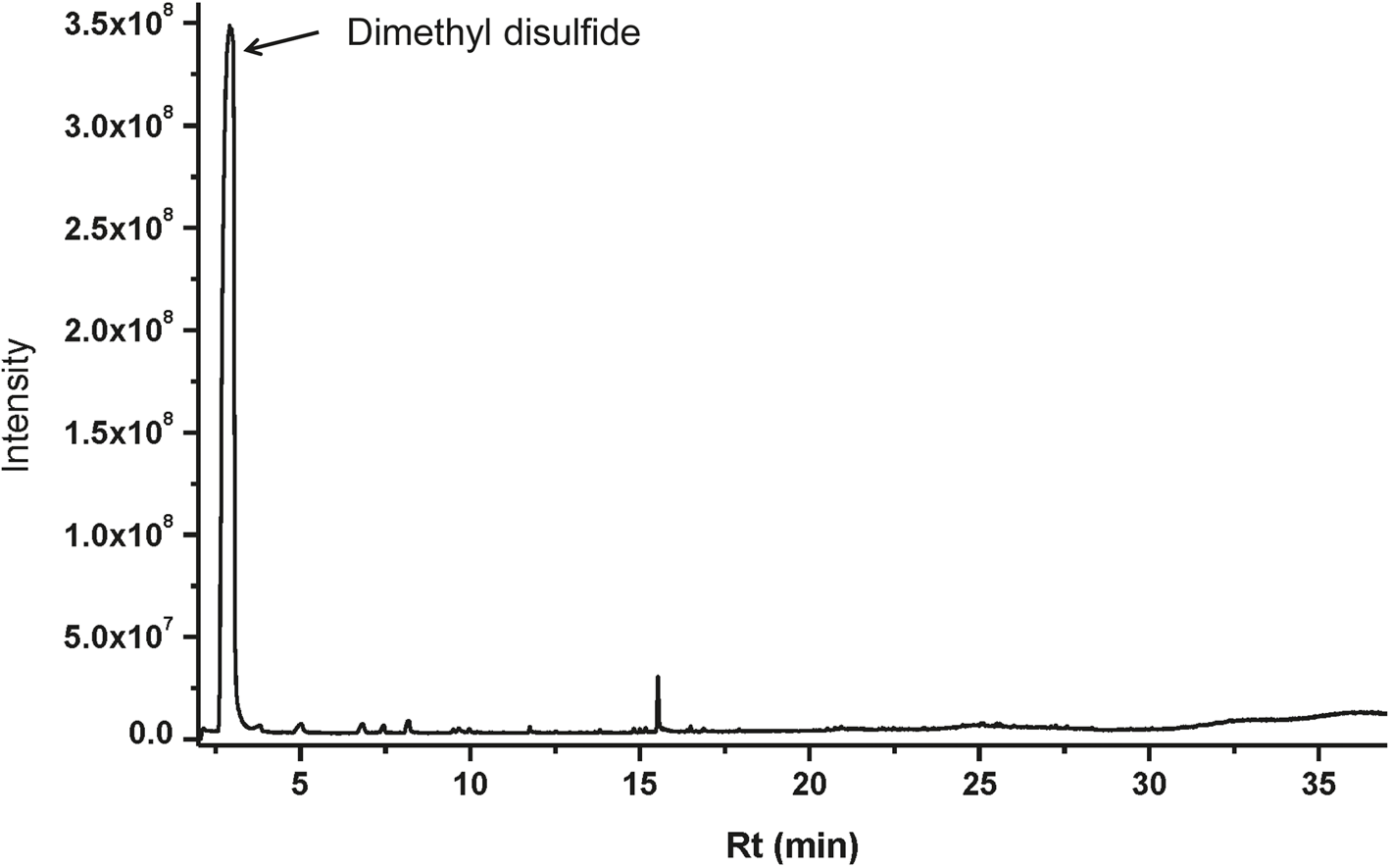
GC-MS analysis of volatiles emitted from strain Pt-3 in NA medium

**Fig. 6:**
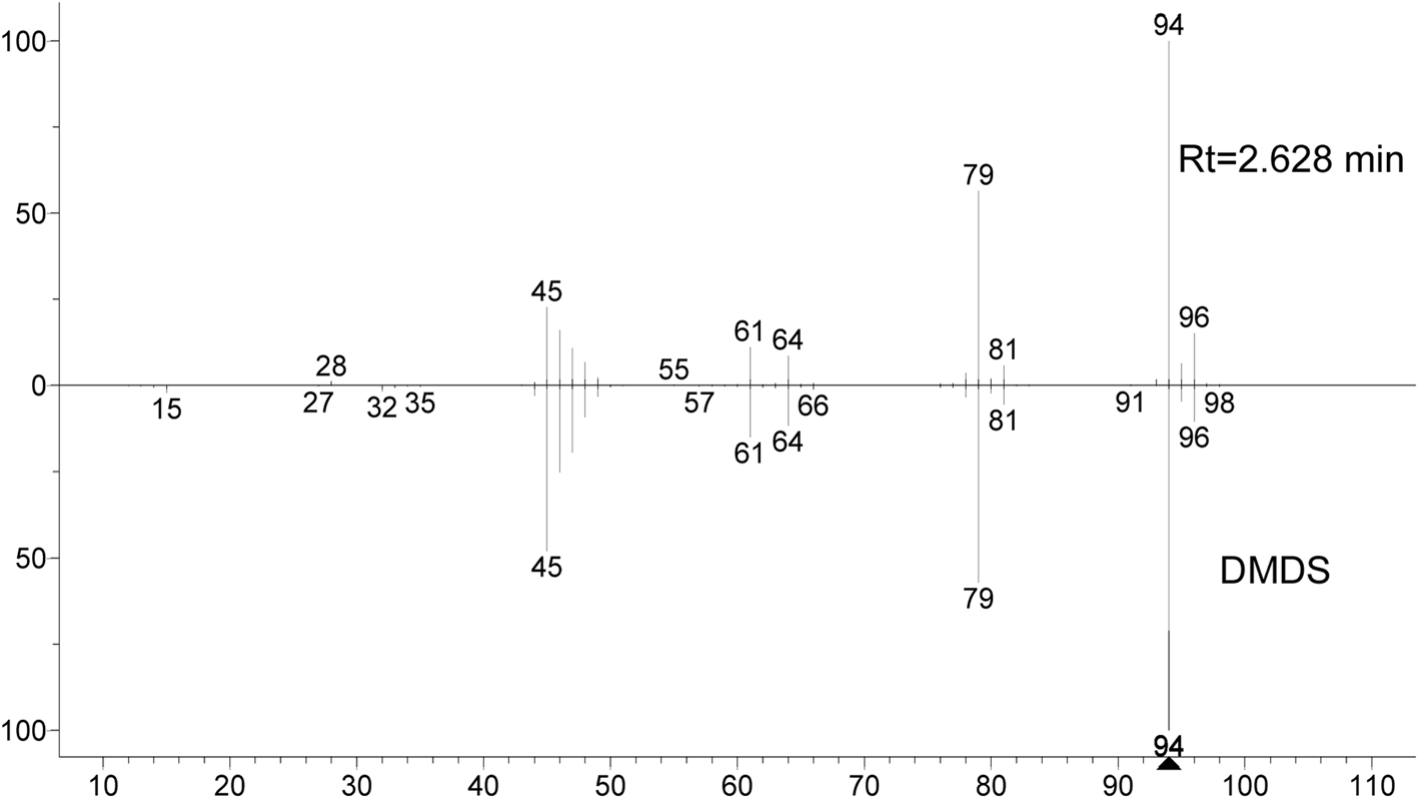
Comparison of mass spectrum of Pt-3 at Rt = 2.628 min and dimethyl disulfide in NIST 17 MS spectral database

The VOCs produced by strain Pt-3 were enriched by SPME equipment, then injected into GC-MS/MS system for further identification. Only one abundant compound was detected in the chromatogram of Pt-3 VOCs during 35 min (Fig. 5). The molecular weight for the compound was 94 Da (D) and showed great similarity (> 95%) to DMDS in NIST11.0 database. The mass peak for the fragments was similar to DMDS under same EI sources (Fig. 6). Additionally, the retention time of detected compounds was 2.628 min which was same to the standard DMTS. These results finally proved that DMDS was the predominant VOC produced by Pt-3.

### Ultra-structure analysis of fungal strain affected by Pt-3 VOCs

**Fig. 7:**
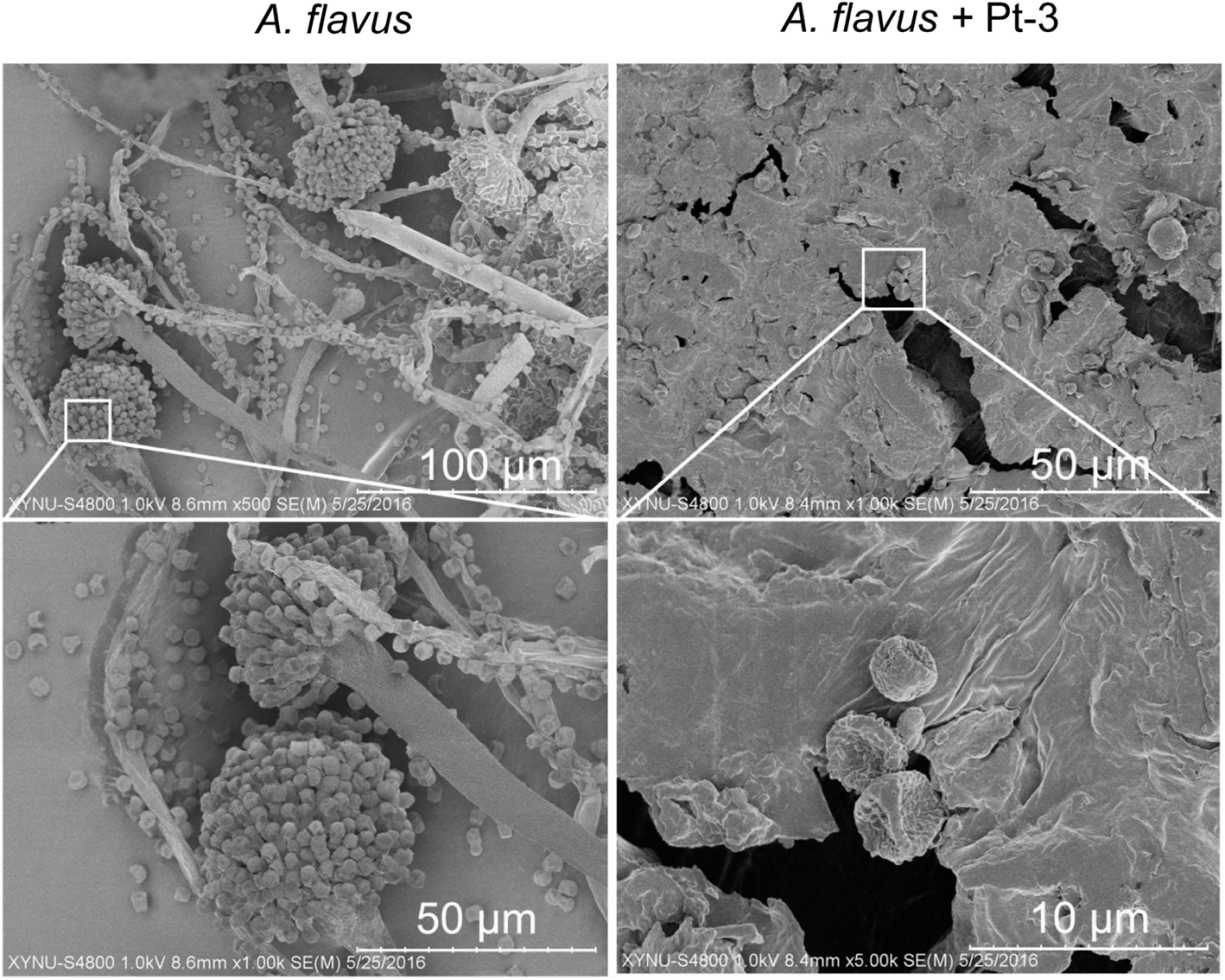
Ultra-structure analysis of *A. flavus* cells infected on peanuts affected by volatiles from Pt-3 under scanning electron microscope

*A. flavus* conidia inoculated on peanuts surface was challenged with Pt-3 for 5 days, the fungal cells on peanuts coat were analysed under scanning electron microscopy (SEM). In control treatment, the conidia can germinate into hyphae and formed conidiophore. Amounts of fresh conidia were produced on conidiophore, and spread over the peanuts coats. Whereas, the *A. flavus* conidia in Pt-3 treatment were severely damage. The conidia can not germinate to hyphae, and showed severely depressed structure (Fig. 7).

## Discussion

Phosphate is one of the most important fertilizers to plant during the whole growth stage. But, the content of active phosphate is seriously deficient in soil of China [4]. Moreover, 95% of phosphate fertilizer applied to soil in season is fixed by metal ions, results in soil compaction and erosion. Hence, the urgent things for soil protection are to alleviate the use of soil fertilizer and improve the content of active phosphate.

It is reported that microbes in soil play important roles in improving soil fertilizer [1]. *Serratia marcescens* as traditional soil bacteria shows positive P-solubilizing activity. Mohamed reported that the *S. marcescens* PH1 and HP2 can form halo zone in solid medium with diameter of 1.1 cm (SI = 3.2) and 0.9 cm (SI = 2.8) respectively [20]. *S. marcescens* CTM 50650 isolated from the phosphate mine also showed P solubilizing activity. The soluble P concentration reached 500 mg.L^− 1^ in liquid medium [7]. *S. marcescens* NBRI1213 exhibited maximum P solubilizing activity of 984 mg.L^− 1^ in liquid suspension [19]. These work indicates that *S. marcescens* with great P solubilizing activity in medium or liquid conditions. But, no P solubilizing activity of these strains are reported in soil conditions. In our current work, *S. marcescens* Pt-3 isolated from tea rhizosphere not only shows active phosphate solubilizing activity in medium, solution and soil conditions, it can also produce abundant antifungal volatiles and greatly inhibit the growth of seven important fungal pathogens. Pt-3 and associated volatiles will provide novel strategies for production of valid bioactive microorganism fertilizer.

In the soil inoculated with Pt-3, the soluble P concentration increases from 2.25 to 3.98 mg.kg^− 1^ during 24 d, although, the P-solubilizing activity of Pt-3 in soil is weak, which is similar to other microbes in soil condition [12]. Take *B. cereus* YL6 as an example, the content of soluble P in the soil is 5.50 mg/kg, which is higher than that in control treatment (4.70 mg/kg). Even weak P-solubilizing activity of YL6 in soil, it really can promote the root growth of Chinese cabbage plants in field [12]. In previous work, we also proved that *Burkholderia cepacia* WY6-5 with weak P- solubilizing activity in soil, can also promote the growth of maize seedling [14]. Soil is a complex interaction environment. Complex compositions and metal ions in soil may interact with soluble P which may further be transferred into insoluble one [2, 21]. Additionally, with the extending of incubation time, the activity and concentration of microbes were changed. That can further affect the P solubilizing activity. Thus, soil compositions as important roles affect the P solubilizing activity of microbe in field. The candidate interactions among microbe, Phosphate and environment should be elucidated in further studies [2, 9].

In our previous work, we proved that volatiles produced by microbe can greatly inhibit the growth of important fungal pathogens, and may further control soil-borne pathogens such as *Shewanella algae* [22], *Enterobacter asburiae* [23], *Alcaligenes faecalis* [24] and *Staphylococcus saprophyticus* [22]. The effective volatiles were identified as dimethyl trisulfide, 1-Pentanol and Phenylethyl Alcohol, methyl isovalerate, 3,3-dimethyl-1,2-epoxybutane respectively. Whereas, the characters of volatiles from *Serratia marcescens* are still unknown till now. Our current work innovatively proved that *Serratia marcescens* Pt-3 can produce volatile dimethyl disulphide, effectively inhibited the growth of seven important phyto-pathogens, and severely damaged fungal cell structure. Additionally, some evidence proved that dimethyl disulfide produced by microbe can spread long distance and greatly inhibit the soil borne pathogens including nematode, *Verticillium dahlia*, *Rhizoctonia solani* and *Cladosporium* spp. [25]. The compounds can also induce systematic resistance and promote the growth of plant in field [26].

*Serratia marcescens* Pt-3 is an efficient phosphate solubilizing bacterium as well as a producer of volatile dimethyl disulfide that showed broad and effective antifungal activity to seven important fungal pathogens. Therefore, *Serratia marcescens* Pt-3 and the produced dimethyl disulfide with multiple functions, which can be used as effective bio-active agents in controlling plant disease and increasing soil fertility.

## Conclusion

*Serratia marcescens* Pt-3 with P-solubilizing and antifungal volatile dimethyl disulfide production activity will provide novel agents for the solving of fertility reduction and pathogen infection in soil.

## Materials and methods

### Microbes and plants

Bacteria Pt-3 was isolated from tea rhizosphere in Xinyang, Henan province, China. Seven important phytopathogens including *Aspergillus flavus, Fusarium graminearum, Alternaria alternata, Magnaporthe oryzae, Aspergillus fumigatus, Colletotrichum graminicola* and *Botrytis cinerea* were isolated from diseased plants and stored in our lab [23, 24]. Peanut (cultivar Silihong) and maize (cultivar Kunyu) seeds were purchased from supermarket. The application of these seeds in our test was permitted in China, and complied with local legislation.

### Isolation of microbe with P-solubilizing activity

Soil samples collected from tea rhizosphere about 10 cm in depth were stored at 4 °C, and used in less than 4 days. For bacteria screening, 1 g soil was placed in 2 ml tubes containing 1 ml sterilized water. The suspension was mix well on vertex for 5 min, then serially diluted to 10^− 6^. One hundred microliters of dilution was spread on NA medium, and cultured at 37 °C for 48 h. The bacteria clones with different phenotypes appeared in NA medium were picked out and streaked on a new NA medium for further tests.

Two kinds of phosphate medium were used in the tests for screening P-solubilizing bacteria. The inorganic P medium contained (NH_4_)_2_SO_4_ 0.5 g, MgSO_4_ 0.3 g, NaCl 0.3 g, Ca_3_(PO_4_)_2_ 8.0 g, glucose 10.0 g, 11% MnSO_4_ 1 mL, 1% FeSO_4_ 1 mL, agar 20 g, 1000 ml distilled water, pH = 7.2. The organic P media included (NH_4_)_2_SO_4_ 0.5 g, NaCl 0.3 g, KCl 0.3 g, CaCO_3_ 5 g, 11% MnSO_4_ 1 mL, 1% FeSO_4_ 1 mL, Soybean lecithin 0.8 g, yeast extraction 0.8 g, agar 20 g, 1000 ml distilled water, pH = 7.2. The medium was autoclaved at 121 °C and 1.01 MPa for 30 min, cooled to room temperature and poured into petri dishes (9 cm in diameter) with 20 ml in each respectively. One medium hole was punched out by a manual disk puncher with a diameter of 5 mm. Obtained microbe suspension (20 μL) collected from NA medium was inoculated into each hole in the medium (organic and inorganic P media). All inoculated media were cultured at 30 °C and darkness for 5 days. Bacterial clones with clear halo zone in phosphate media were considered as PSB and selected for further use. The P-solubilizing activity of each microbe was conducted for two times, and the phosphate solubilizing index (PSI) was calculated as the following equation [20, 21].

$$\mathrm{PSI}=\left(\mathrm{colony}\ \mathrm{diameter}+\mathrm{halozone}\ \mathrm{diameter}\right)/\mathrm{colony}\ \mathrm{diameter}\times 100.$$

### P-solubilizing activity of Pt-3 in liquid broth and soil

Pt-3 was cultured in NA medium for 48 h. The fresh bacteria bodies were collected and adjusted to 10^8^ cfu/mL in sterilized water. To analyze the P-solubilizing activity of Pt-3, 100 μL of bacteria suspension was inoculated into 40 mL of the liquid inorganic P medium ((NH4)2SO4 0.5 g, MgSO4 0.3 g, NaCl 0.3 g, Ca3(PO4)2 8.0 g, glucose 10.0 g, 11% MnSO4 1 mL, 1% FeSO4 1 mL, 1000 ml distilled water with pH 7.2) in 100 ml flask. The media without bacteria were used as control. All flasks were placed at 30 °C and 100 rpm for 20 days. One milliliter of suspension was collected from the flask every 6 days. The suspension was centrifuged at 8000 rpm for 15 min, and the supernatant was transferred into a new tube for PO_4_^3−^ analysis. The soluble PO_4_^3−^ in the supernatant cultures was determined with a Segmented Continuous Flow Analyzer (Futura, Alliance, France) at a wavelength of 420 nm [2].

To determine the P-solubilizing activity of bacteria in soil, soil sample was collected from tea rhizosphere of 10 cm in depth, dried at room temperature and sieved through 40-mesh screen. 1.70 kg soil was filled into a plastic bag, 15% (v/w) of inorganic P medium was added into each soil sample and autoclaved at 121 °C for 20 min (three times). Strain Pt-3 at OD 0.354 was inoculated to soil samples at 5% (v/w). Sterilized water was used as control. Each test was conducted for three times, and all soil samples were cultured at 28 °C and darkness for 30 days. The soil (10 g) was collected from each bag every 6 days and used for PO_4_^3−^ quantitative analysis.

The soil samples were re-suspended in 10 mL sterilized water, mixed at vortex for 10 min, and centrifuged at 3500 rpm for 10 min. The supernatant was then filtered through a 0.45 μm filter. Released PO_4_^3−^ was measured using the method described above. The P-solubilizing activity of Pt-3 in liquid and soil conditions were conducted for two times with three replication in each.

### Plant growth promoting activity of Pt-3 on maize seedling

Soil sample was collected from the rhizosphere of tea plant about 5-10 cm in depth. The soil sample was dried at room temperature, sieved through a mesh of 2.00 mm in side length. Then, the soil was filled in plastic bags and autoclaved at 121 °C and 30 min for 3 times. After cooling to room temperature, the liquid P solubilizing medium was added into the soil samples (relative to the soil 15% v/w). Then, the soil was equally separated into six parts, and placed into six pots (19.2 cm in diameter, 14.2 cm in height) with 1.8 kg in each. Pt-3 suspension was inoculated into three pots and blended well with a stirring rod. The other three pots inoculated with sterilized water were used as control.

Maize seeds were surface sterilized in 75% ethanol for 3 min, rinsed and soaked with sterilized water for 30 min, then placed on moistened filter paper for germination. The germinated seeds with same bud length were picked, and sowed into the pots filled with soil. Three pots were used in each treatment, and four seeds were planting in each pot. All pots were maintained in the open air and occasionally watered with sterile water for 30 days. Finally, the maize seedlings were obtained, and the parameters (including plant height, leaf and root length, leaf number and width, dry weight) were measured.

### Molecular identification of strain Pt-3

The genomic sequence of Pt-3 was extracted by Tris-HCl (Amresco) and EDTA (Amresco) methods [3]. The 16S rRNA sequences were amplified by PCR methods using the universal primers 27F (AGAGTTTGATCCTGGCTCAG) and 1541R (AAGGAGGTGATCCAG CCGC) [27, 28]. The PCR conditions used were as follows: initial denaturation at 94 °C for 5 min; followed by 30 cycles of 94 °C for 30 s, 55 °C for 30 s, and 72 °C for 40 s; then 72 °C for 10 min. PCR products were analyzed by gel electrophoresis and purified for sequencing analysis. The sequences were aligned in GenBank database. The obtained 16S rRNA sequences of bacterial strain with similarity over 95% were selected for further phylogenic tree analysis. Twelve strains including Pt-3, *Serratia plymuthica* BSP25, K-7, UBCF13, T237, ZC06-1, AG2105, SFC20131227, *Serratia liquefaciens* CPAC53, ATCC27592, and *Serratia entomophila* Mor4.1, DSM12358 were used for phylogenetic tree analysis. The tree was constructed using the MEGA software with the neighbor-joining method [2, 29, 30].

### Biochemical and biophysical analysis of strain Pt-3

Strain Pt-3 was cultured on commercial BUG medium (Biolog, Hayward, CA) at 28 °C for 24 h. The fresh bacteria clone was inoculated into solution A (Biolog, Hayward, CA) and adjusted to percent transmittance 95% through Biolog Turbidimeter (Biolog, Hayward, CA). The bacteria suspensions were mixed well and distributed into Microplate (Biolog, Hayward, CA) with 100 μL in each hole. The plates were cultured at 28 °C for 12 h. The biochemical results of Pt-3 were recorded through Biolog Gen III system (Biolog, Hayward, CA) [24].

### Broad spectrum antifungal activity of strain Pt - 3

The antifungal activity of strain Pt-3 was analyzed through FTF dual cultural tests in two Petri dishes. Pt-3 was cultured on the surface of NA plate at the bottom. Fresh fungal strains were inoculated to the center of PDA plate in the top. The PDA plate was placed face-to-face on the top of NA plate. The PDA plate with fungal co-cultured with NA plate without Pt-3 was used as control. All plate pairs were sealed with tapes and cultured at 28 °C and darkness for 7 d. The inhibition of Pt-3 against pathogenic fungi was conducted for two times, and the inhibitory rate was calculated based on the following formula: Inhibitory rate (%) = (mycelia diameter of the control- mycelia diameter of the Pt-3) / diameter of the control × 100.

### Effect of active charcoal on inhibitory activity of Pt-3

Pt-3 could inhibit the growth of fungal in face-to-face cultural tests. To further prove the antifungal activity of volatiles from Pt-3, active charcoal (5 g) was added in the tests to verify the fumigation efficacy of VOCs from Pt-3. Here, four pairs of Petri dishes were used: (a) *A. flavus* inoculated to the center of PDA plate. (b) Pt-3 on NA against *A. flavus* inoculation on PDA. (c) Active charcoal in Petri dish against *A. flavus* on PDA. (d) active charcoal, Pt-3 on NA against *A. flavus* on PDA. All plates were incubated at 28 °C for 5 days [22]. Similarly, the diameters of the fungi on PDA plate were recorded and the inhibitory rate was calculated as before.

### Identification of VOCs from Pt-3 by GC-MS

VOCs from Pt-3 showed broad antifungal activity. To further identify the predominant compounds in VOCs, the VOCs was enriched by Solid-phase micro-extraction (SPME), and further identified by Gas Chromatography tandem Mass Spectrometry (GC-MS/MS). Pt-3 (100 μl, 10^8^ cfu/ml) was spread on NA medium surface (40 ml) in a 100 ml flask. The flask was sealed with plastic membranes. NA medium without bacteria was used as control. All flasks were cultured at 28 °C and darkness for 48 h [22]. Flasks were transferred to a pre-heated 40 °C water-bath and allowed to equilibrate for 30 min. The adsorption head of SPME was injected into the flaks and absorbed for 40 min, then used for GC-MS analysis [31].

For GC-MS analysis, the column was DB-5 MS capillary column (30 m × 0.25 mm ID, 0.25 mm thickness film). Helium was used as carrier gas at the flow rate of 1 ml/min. The inlet temperature was 250 °C. The oven temperature was set as follows: 40 °C for 3 min, gradually increased to 160 °C at the rate of 3 °C/min, and maintained for 2 min. Finally, the temperature was increased to 220 °C at 8 °C/min, and lasted for 3 min [32, 33]. For MS, the spectrometers were operated in electron-impact (EI) mode, the scan range was 50–550 amu. The inlet, ionization source and quadrupole temperature were 300, 230 and 150 °C, respectively. The compounds identified in the spectrometry profiles of Pt-3, not presented in control samples were considered to be the final analytes. The compounds were aligned in National Institution of Standards and Technology (NIST 11) database [32, 33]. The retention time and mass spectrum of identified compounds was compared with commercial standards for final qualitative analysis.

### Scanning electron microscope analysis

*A. flavus* mycelium was inoculated to the center of PDA medium and cultured at 28 °C for 5 days. Fresh conidia on PDA surface were washed off with sterilized water and filtrated through two layers of gauze, and diluted to a concentration of approximately 10^5^/ml for peanuts inoculation tests [22].

Peanut seeds (100 g) of uniform size were placed in 250 ml flasks and autoclaved at 121 °C and 1.01 MPa for 20 min. Ten ml sterilized water containing *A. flavus* conidia (10^4^ cfu/ml) was added to the flask, mixed well and adjust to water activity (a_w_) 0.9. The peanut seeds inoculated with *A. flavus* were divided in two petri dishes. One dish was challenged with Pt-3 (grown on NA plate) with face-to-face method. The other dish challenged with NA medium was used as control. All Petri dishes were incubated at 28 °C for 5 days. The peanut seeds were fixed in 0.1% osmic acid for 1 h. Then, a small piece of the peanut coat about 3 × 3 mm was peeled off, affixed to stubs, coated with gold and investigated through scanning electron microscope [23, 31].

### Data analysis

All experiments were carried out at least in twice and results were reported as means ± standard deviations. The significant differences were determined using Student’s T tests (*p* < 0.05) following one-way analysis of variance (ANOVA). The statistical analysis was performed using SPSS 16.0 software (SPSS Inc., Chicago, USA).

## Data Availability

All data generated or analyzed during this study are included in this published article. The 16S rRNA sequence of Pt-3 generated and analysed during the current study are available in the GenBank repository with accession number OL636198.
